# Culturing primary neurons from rat hippocampus and cortex

**DOI:** 10.1042/NS20180207

**Published:** 2019-04-26

**Authors:** Madhusmita Priyadarshini Sahu, Outi Nikkilä, Seija Lågas, Sulo Kolehmainen, Eero Castrén

**Affiliations:** Neuroscience Center, Helsinki Institute of Life Science HiLIFE, University of Helsinki, Helsinki 00290, Finland

**Keywords:** cerebral cortex, hippocampus, primary neuron

## Abstract

Primary neurons from rodent brain hippocampus and cortex have served as important tools in biomedical research over the years. However, protocols for the preparation of primary neurons vary, which often lead to conflicting results. This report provides a robust and reliable protocol for the production of primary neuronal cultures from the cortex and hippocampus with minimal contribution of non-neuronal cells. The neurons were grown in serum-free media and maintained for several weeks without any additional feeder cells. The neuronal cultures maintained according to this protocol differentiate and by 3 weeks develop extensive axonal and dendritic branching. The cultures produced by this method show excellent reproducibility and can be used for histological, molecular and biochemical methods.

## Introduction

A primary neuron culture from embryonic rodent hippocampus or cortex has been one of the most fundamental methodologies for modern neurobiology. Primary neurons can be easily cultured and over a few days or weeks differentiated into neurons with clearly separable axons, dendrites, dendritic spines and synapses. By modifying the culture medium and conditions, numerous factors responsible for directing different aspects of neuronal survival, differentiation and phenotype have been revealed [1]. Earlier publications have more or less thoroughly described protocols for cultures of primary neurons over the years. Original methods have used serum to support neuronal survival and differentiation [2] but more recently culture methods using defined media without serum have been introduced [3–6]. Glial cells provide critical support to cultured neurons [7–9]. Several methods to keep excess glial proliferation in check or to prevent mixing between neurons and glia have been described [10].

 One of the disadvantages of primary culture is that they do not divide in culture and need to be generated from embryonic or early postnatal brains every time. Moreover, successful dissection and preparation of cultures require substantial skill and experience. Over several decades, cell lines have been discovered and created that mimic many or most of the features of primary neurons [11,12]. More recently, differentiated stem cells from rodents [13] or humans [14] have been introduced as alternatives for primary cultures, but none of these have replaced embryonic primary neurons from their position as a gold standard for neuronal cultures.

Neuronal cultures, however, vary vastly depending on source, age of derivation and culture conditions. Results obtained by a culture protocol used in one lab may not be reproducible in another lab, which adds to the ongoing discussion about reproducibility crisis. We have over more than a decade developed and refined a culture protocol of primary neurons derived from E17–18 rat embryos, which has been successfully used in several publications [15,16]. Here, we describe the protocol, necessary materials and methods as well as the characteristics of neurons derived through it in detail and share the protocol with other groups with the aim to promote reproducibility and rigor.

## Materials and methods

### Animals

Pregnant female Wistar rats were obtained from Envigo (Harlan Labs, U.K.). The plug date of the female rats was marked E0. All embryos staged at E17–18 from the female rats were used in the experiments. The embryos were staged according to the Witschi Standard Rat Stages. The average litter size was 9 pups per female rat. Animals were kept in standard conditions (temperature 22 ± 1°C, 12-h light/dark cycle). Food and water were available *ad libitum*. All the experiments were performed at the Animal Unit, Biomedicum, University of Helsinki. The procedures were followed according to institutional guidelines by the University of Helsinki internal license number: KEK17-016.

### Composition of solutions (for material source, refer Table 1)

1. Phosphate-buffered saline (PBS) buffer pH = 7.4
  - 80 g NaCl,
  - 0.2 g KCl,
  - 14 g Na_2_HPO_4_ × 2H_2_O,
  - 2 g KH_2_PO_4_.
    Make upto 1 l with Milli-Q H_2_O and autoclaved at 121°C for 20 min.
2. Preparation medium, pH = 7.2
  - HBBS (Hank’s balanced salt solution),
  - 1 mM sodium pyruvate,
  - 10 mM HEPES.
3. Dulbecco’s modified Eagle’s medium (DMEM) ++
  - DMEM,
  - 10% fetal bovine serum,
  - 1% l-glutamine,
  - 1% penicillin–streptomycin.
4. The stock Papain buffer stored at −20°C
  - 1 mg DL-Cysteine HCl,
  - 1 mg bovine serum albumin (BSA),
  - 25 mg Glucose in 5 ml PBS.
5. Papain solution
  - 0.5 mg papain,
  - 10 μg DNase I in 5 ml Papain Buffer.
6. Trituration medium
  - 10 μg DNase I in 10 ml Preparation medium as above.
7. Growing medium
  - Neurobasal medium,
  - 2% B27 supplement,
  - 1% L-glutamine,
  - 1% penicillin–streptomycin.
8. Poly-l-lysine working solution was made as 1:10 dilution of stock Poly-l-lysine in Milli-Q H_2_O.
9. 4% Paraformaldehyde (PFA)
  - 40 g PFA in 1 l PBS [1].
    The final solution was filtered with a Whatman filter paper (pore size 45 mm).
10. PBST
  - 0.3% Triton X-100 in PBS.
11. Blocking buffer
  - 1% BSA,
  - 4% normal goat serum,
  - 0.3% Triton X-100 in PBS.
12. Primary antibodies used were glial fibrillary acidic protein (GFAP), neuronal nuclei (NeuN) and microtubule-associated protein 2 (Map2) (Table 1) at 1:1000 for GFAP and NeuN and 1:10000 for Map2 dilution in the blocking buffer [11].
13. Secondary antibodies were goat-anti-rabbit 647, goat-anti-mouse 568 and goat-anti-chicken 488 diluted 1:1000 in blocking buffer.

**Table 1:** Details of the material source

Reagents	Equipments and surgical instruments	Other materials
NaCl (31434, Riedel)	Dissecting/stereo microscope (EZ4 HD, Leica)	Petri dish 90 mm (101RT/C, Thermo Scientific)
KCl (31248, Riedel)	CO_2_ Incubator (Heracell, Heraeus)	Petri dish 60 mm (628102, Greiner)
Na_2_HPO_4_ × 2H_2_O (0326, J.T. BAKER)	Centrifuge 5810 (Eppendorf)	Petri dish 35 mm (627102, Greiner)
KH_2_PO_4_ (4871, Merck)	Scissors (RU 1003-14 Rudolf)	Multidish four-well plate (176740, Thermo scientific)
HBBS (14170088, Gibco)	Spring scissors (15004-08 Fine Science Tools)	Superfrost slides (J1800AMNT, Thermo Scientific)
100 mM sodium pyruvate (11360039, Gibco)	Forceps (11000-13 Fine Science Tools)	Coverslips (Round 13 mm, Thermo Scientific)
1 M HEPES pH 7,2 (101926, ICN)	Curved forceps (11271-30/Dumont#7 Fine Science Tools)	C-Chip Disposable Hemocytometer (Bürker, LabTech)
DMEM (BE12-614F, BioWhittaker Lonza)	Straight forceps (11295-10/Dumont#5 Fine Science Tools)	Microscope slides (ECN 631-1551, VWR)
Papain (P-4762, Sigma)	15- and 50-ml Falcon tubes (CLS430791 Sigma)	
DNase I (D4527, Sigma)		
dl-Cystein HCl (C-9768, Sigma)		
BSA (A7638, Sigma)		
d-(+)-Glucose anhydrous (49139, Fluka)		
PFA (1157, J.T. BAKER)		
Poly-l-lysine (P4707, Sigma)		
Normal goat serum (16210-064, Lifetechnologies, Gibco)		
Triton X-100 (93426, Fluka)		
Anti-GFAP antibody (12389, Cell Signaling Technology)		
Anti-NeuN antibody (MAB377X, EMD Millipore)		
Anti-Map2 antibody (ab5392, Abcam)		
Goat anti-rabbit 647 (A21245, Life Technologies)		
Goat anti-mouse 568 (A11004, Life Technologies)		
Goat anti-chicken 488 (A11039, LifeTecnologies)		
Mounting media with DAPI (ab104139, Abcam)		
Trypan Blue (T8154, Sigma)		

### Extraction procedures

**Figure 1:**
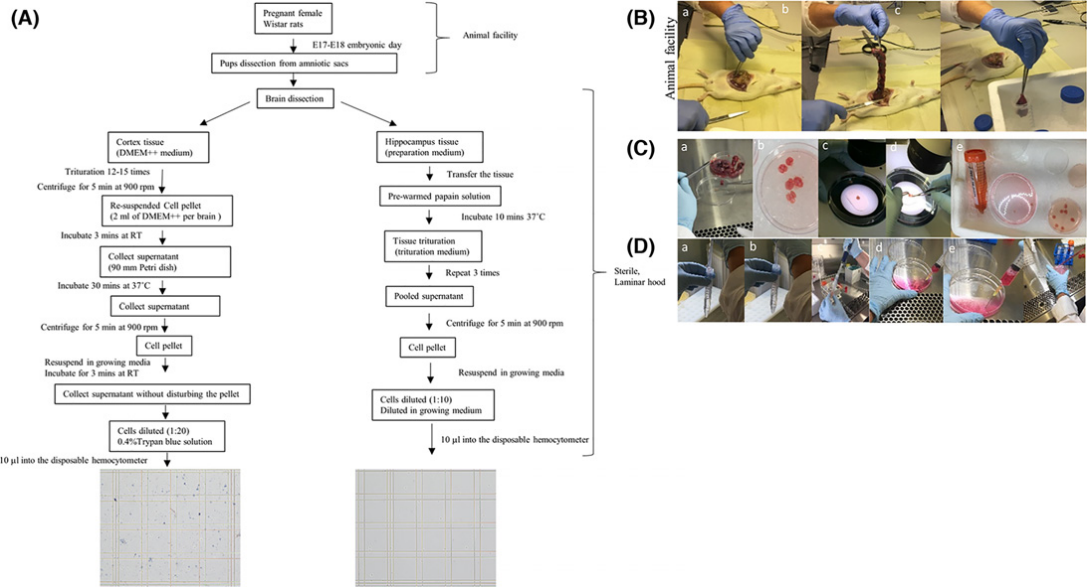
The procedure for extracting neuronal cells from the intact animal tissue (**A**) Flowchart summarizing the complete procedure. (**B**) This part of the procedure was performed in the animal facility (a) opening the visceral cavity of the rat, (b) extracting the pups, (c) collecting the pups into sterile PBS. (**C**) This procedure was performed in the sterile laminar hood. (a–c) Extraction of the brain from the pups, (d,e) dissecting the cortex and hippocampus from the brain. (**D**) Trituration of the tissue to produce homogeneous cells, (a–c) hippocampal neurons and (d–f) cortical neurons.

The pregnant female Wistar rats were terminally anesthetized with carbon dioxide (CO_2_) in a CO_2_ euthanasia chamber. Abdominal skin was washed with 70% ethanol followed by an incision to cut and open the peritoneal cavity (Supplementary video S1). Amniotic sacs were exposed with fine scissors and embryos were taken out from the uterus. These embryos were transferred to a 50-ml polypropylene Falcon tube with 30 ml of PBS on ice. The embryos were transferred to a sterile laminar hood where further processing took place. The heads were decapitated by scissors and immediately transferred to ice-cold PBS stored on ice. Brain dissection was performed in 10-ml preparation medium [2] on a 60-mm Petri dish on ice. For precise identification of the brain structures, the dissection of cortex and hippocampus were performed under a Leica stereo microscope at room temperature (RT). Dissection is demonstrated step by step in the supplementary video files (Supplementary videos S2–S5) and in Figure 1. Hippocampal and cortical neurons were prepared separately according to the following instructions. All the experiments were carried out under sterile conditions.

### Cell preparation from hippocampus tissue

**Figure 2:**
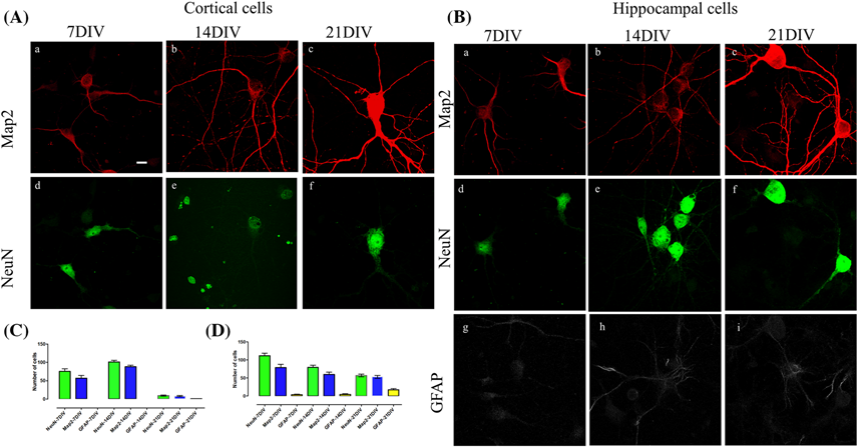
Cultured cortical and hippocampal primary neurons stained at three different time points for neuronal markers Map2, NeuN and GFAP (**A**) (a–f) Cortical neurons at 7 DIV (a,d), 14 DIV (b,e) and 21 DIV (c,f). (**B**) Hippocampal neurons at 7 DIV (a,d,g), 14 DIV (b,e,h) and 21 DIV (c,f,i). (**C**) Cell counting in cortical neurons using markers for neurons (NeuN), astrocytes (GFAP) and a dendritic cell marker, Map2. (**D**) Cell counting of the representative markers in hippocampal neurons. The neurons are stained with NeuN (green), Map2 (red) and GFAP (gray). Scale bar = 10 µm. Data represented as mean ± SEM. Abbreviations: DIV, days *in vitro*.

1. The dissected hippocampi were transferred into 10 ml of preparation medium [2] on a 35-mm Petri dish on ice.
2. The hippocampi were transferred using a fire-polished glass Pasteur pipette into a 15-ml polypropylene Falcon tube with 5 ml of papain buffer [4] with added papain solution [5] at 37°C. The papain buffer was pre-warmed for 15 min in a water bath.
3. The tissue was incubated for 10 min at 37°C. After the tissue had sunk to the bottom, excess papain solution was discarded using a glass pipette.
4. Pipette 3 ml of trituration medium [6] to the tissue into the same tube.
5. The cells were triturated in the trituration medium [6] with a fire-polished glass Pasteur pipette for ten times. The remaining non-dissociated tissue was allowed to sink to the bottom of the tube for approximately 30–60 s. The supernatant was collected by a fire-polished glass Pasteur pipette and transferred to a fresh 15 ml Falcon tube without disturbing the un-dissociated tissue.
6. The trituration was repeated for three times with a fire-polished glass Pasteur pipette.
7. The pooled supernatant with the dissociated tissue from each trituration step (approximately 9 ml) was centrifuged for 5 min at 900 rpm/154×***g*** in Centrifuge 5810 (Eppendorf) at RT.
8. The clear supernatant was discarded.
9. The remaining cell pellet was re-suspended in 1 ml of fresh growing medium at RT [7].
10. The hippocampal cells were diluted to 1:10 (5 μl of cells + 45 μl growing media). This gentle dissociation method results in hippocampal cells with very few dead cells or debris. Therefore, diluted cells were directly used for cell counting. Ten microliters of the diluted cells were transferred into a disposable hemocytometer by a pipette. For cell counting, cells in four fields of the hemocytometer were counted using a manual cell counter under a Leica stereo microscope and averaged.
11. Hippocampi from ten embryos typically yields 5–6 million neurons.
12. Representative examples of different stages of cell differentiation are shown in Figure 2. For immunostaining, cells were plated on to coverslips within a 24-well plate at the density of 26000 cells/cm^2^. The cells were grown at 37°C, 5% CO_2_ humidified incubator (Heracell, Heraeus) for the indicated times (Figure 2 ) before fixation and staining.

### Cell preparation from cortex tissue

1. Cortices were dissected and tissues were allowed to recover in 10 ml DMEM++ [3] on a 90-mm Petri dish on ice, until the dissection of all brain samples was completed. All dissected brain tissues were placed on to the same plate.
2. The cortical tissues were triturated in DMEM++ [3] in the same Petri dish plate with a 20G needle and 10 ml syringe for 12–15 times (Supplementary Video S6). After trituration, the cell suspension was transferred to a 15-ml Falcon tube.
3. The triturated tissue was centrifuged for 5 min at 900 rpm/154×***g*** at RT. After centrifugation, the cloudy supernatant was carefully removed with a pipette and discarded.
4. The cell pellet was re-suspended in 0.5 ml of DMEM++ [3] per each dissected brain (for example, 2.5 ml for cortices from five embryos) per brain sample. Incubate the tube for 3 min.
5. Pipette 7.5 ml of DMEM++ on to uncoated 90 mm Petri dishes. Pipette 2.5 ml of the supernatant from the previous step (representing five brains) on to each plate.
6. The plates were incubated at 37°C, 5% CO_2_ humidified incubator for 30 min. During this incubation, glial cells and other unwanted debris adhere to the bottom of the plate and the cortical cells are also recovering from the trituration.
7. Pipette 7.5 ml of DMEM++ on to uncoated 90 mm Petri dishes. Additionally, pipette 2.5 ml of the supernatant from the previous step (representing five brains) on to each new Petri dish or dishes.
8. The supernatant was carefully removed, without disturbing attached glial cells and unwanted cell debris at the bottom of the plate into a new 15-ml Falcon tube using a pipette. The collected supernatant from the above step was centrifuged for 5 min at 900 rpm/154×***g*** in RT.
9. After centrifugation, the supernatant was discarded and the pellet was re-suspended in 1.5 ml growing medium [7] per each plate (representing five brains) at RT. It was allowed to settle under gravity for 2–3 min to sediment the debris.
10. The cell suspension was removed into a new 15-ml Falcon tube using a pipette.
11. The cortical cells obtained by this method results in a lot of cell debris. Therefore, cell counting for the cortical cells was performed along with 0.4% of Trypan Blue dye. The cortical cells were used at 1:20 dilution (5 μl of cell suspension + 75 μl growing medium + 20 μl Trypan Blue) for cell counting. The cell counting was done similarly as for the hippocampal cells.
12. Cortices from ten embryos typically yield 50–60 million neurons. If the number of embryos is low, the relative yield is typically lower than when the cells are prepared from a large number of embryos.

### Preparation of plates

The multi-well plates (for biochemical assays) or round coverslips (histochemistry) within a 4- or 24-well plate were pre-coated for 18 h with 500 µl of 10 μg/ml Poly-l-lysine [8] at 37°C. The coverslips used for plating cells were sterilized with 96% ethanol followed by drying through the tissue paper to remove excess ethanol from the coverslips. These coverslips were then moved to the wells and air dried for 5 min before pre-coating with Poly-l-Lysine. After at least 18 h (overnight) of incubation and before plating the cells, the plates were rinsed twice with 500 µl of PBS.

### Cell maintenance

The number of cells plated per well varies depending on the downstream processing of the samples. Representative examples of plating densities for different cell types and well sizes are documented in Table 2. Cells were grown at 37°C, 5% CO_2_ in a humidified incubator (Heracell, Heraeus). Half of the growing medium was changed once a week.

**Table 2:** Examples of typical plating densities (number of cells/well)

Type of plate	6-well	12-well	24-well	96-well
Area	(∼9 cm^2^)	(∼4 cm^2^)	(1.9 cm^2^)	(0 cm^2^)
Hippocampal neurons				
- for biochemistry	500000	250000	125000	3000–60 000
- for histology (coverslips)			50000–125000	
Cortical neurons				
- for biochemistry	1000000	500000	250000	3000–60000
- for histology (coverslips)			50000–125000	

### Immunohistochemistry

For immunostaining, we used 26000 cells/cm^2^ on the coverslip in a four-well plate. The cells were grown at 37°C, 5% CO_2_ in a humidified incubator. The cell plating density always depended on the purpose of the experiment, examples of typical cell plating densities are shown in the Table 2. The hippocampal and cortical cells were grown for 7, 14 and 21 days in the humidified incubator at 37°C. Cells were fixed at respective time points with 4% PFA [9] for 15 min followed by a single wash with 200 µl of PBS for 5 min. The liquids were removed by suction pump throughout this procedure.

1. The four-well plates were washed three times for 5 min with 200 µl of PBST [10].
2. The fixed cells were pre-blocked with 200 µl blocking buffer [11] for 1 h at RT.
3. After blocking, 150 µl of primary antibody [12] diluted in the blocking buffer [11] was added to the wells. This was left on a shaker set at 100 rpm at 4°C for at least 18 h.
4. After incubation with the primary antibody, the buffer was removed and cells were washed 3 × 10 min with 200 µl of PBST.
5. The secondary antibody [13] was diluted into 150 µl of blocking buffer and added to the wells. The cells were left for at least 18 h on a shaker set at 100 rpm at 4°C. The plates were covered with an aluminium foil to protect them from light.
6. After the incubation with the secondary antibody, the coverslips were washed for 10 min in 200 µl of PBST followed by two times for 10 min with 200 µl of PBS.
7. The coverslips were held with fine forceps and dipped into 50 ml Falcon tubes containing Milli-Q H_2_O to rinse off the PBS. Before mounting, coverslips were gently dried by touching the edge on to a tissue paper.
8. The cells attached on to the coverslips were mounted on the Superfrost slides with 10 µl of mounting media (Table 1). Two coverslips per slide were mounted and excess of mounted media was cleaned for imaging purposes.
9. The slides were stored in dark, protected from light at 4°C until imaging.

### Imaging

The whole slide imaging was performed using a 20× objective in a Histoscanner (3D HISTEC Ltd., Hungary) at the genome biology unit, Biomedicum Helsinki. The images were analyzed using panoramic viewer software (3D HISTEC Ltd., Hungary). Four coverslips for each stage were scanned. Cell counting was performed using ImageJ software (https://imagej.nih.gov). Cells from a single focal plane were analyzed for an area of 4 mm^2^ per image per coverslip. We analyzed five images per coverslip and four coverslips for each time point was used for imaging. Thus, 20 images for each time point was analyzed.

The higher magnification images were acquired using a 63× objective in a Zeiss LSM 880 confocal microscope, at the Biomedicum Imaging unit, Biomedicum Helsinki. The lasers used were Alexa 488, Alexa 565 and Alexa 647. To minimize cross-talk between different channels, the images were acquired by sequential scanning. The most commonly used algorithm for image acquisition was the maximum projection. The maximum intensity method is useful in extracting and detecting finer structures in a three-dimensional mode [17].

### Statistical analysis

All the data were analyzed using the GraphPad Prism 6 software (La Jolla, CA, U.S.A.). The groups were compared using one-way ANOVA. All the data are represented as means ± SEM.

## Results

The method for culturing primary neurons described here has been developed and used in our lab for over two decades. The quality of neurons has been high and reproducible over the period of time. The overall procedure is outlined in Figure 1 (Figure 1A). The extraction of the pups was performed under semi-sterile conditions within the animal facility (Figure 1B). The brain extraction and dissection were performed in a laminar hood under sterile conditions (Figure 1C). The trituration for hippocampal neurons and cortical neurons was performed separately (Figure 1D). The hippocampal cell preparation typically results in few dead cells and lack of any debris, and they can be used directly in the hemocytometer for cell counting without any staining. In contrast, trituration of cortical neurons is harsher, and the cells therefore need to be stained with Trypan Blue to exclude dead cells.

**Figure 3:**
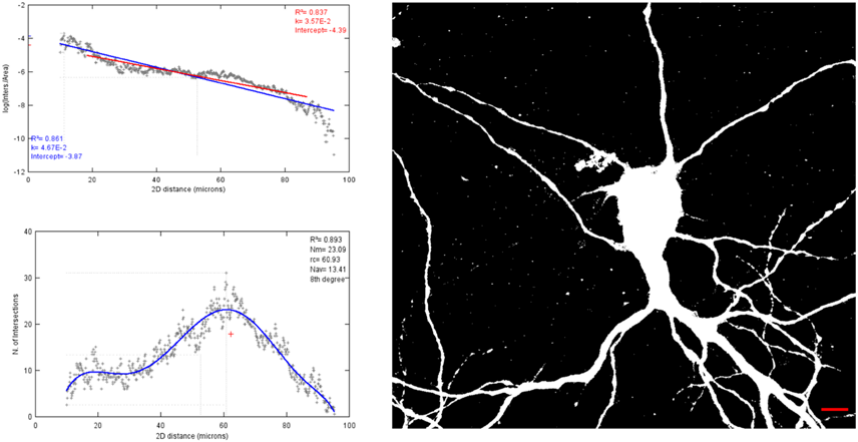
A representative sample image used for Sholl analysis of 21 DIV hippocampal neuron. The graphs show retrieved metrics of the linear Sholl plot for the bitmap image using ImageJ

The markers for cell staining were selected to identify neurons from glial cells if any were present. Furthermore, the level of maturation of the neurons was studied using different cell type markers along with a dendritic marker. The cells were stained for markers such as NeuN (stains neuronal nuclei), GFAP (stains glial cells) and Map2 (stains neuronal somato-dendritic compartment) at three different time points (Figure 2). The density of cells was approximately 26000 cells/cm^2^ for both hippocampal and cortical cells. In the cortical cell preparations, no GFAP positive cells were detected at 7 and 14 days *in vitro* (DIV). At 21 DIV the GFAP cells formed 4% of the total cell population in the primary cortical cells (Figure 2A). The number of cortical neurons sharply decline at 21 DIV. For primary hippocampal neurons, the number of GFAP positive cells was 2% at 7 DIV, 6% at 14 DIV and 28.5% at 21 DIV of the total cell population (Figure 2B). The dendritic branching in both the cells increase from 7 until 21 DIV. As an example of quantitative analysis, a Sholl analysis [18] of hippocampal neuron branching was performed (Figure 3).

## Discussion

The protocol described here is originally based on the protocol described by Brewer and Cotman [4], with some early modifications [19]. When neurobasal and the B27 supplement became available [1], the protocol was adopted accordingly. Over the years, the protocol has evolved with small modifications introduced based on experience. This protocol has been cited in several publications [15,16], but it has never been properly published. We have now described the current protocol in detail and supplemented the protocol with photographs and videos. We hope that this description will help research groups starting to use primary neuronal cultures as a research tool, and perhaps also give useful hints for groups already using these cultures based on their own protocols that typically evolve over years.

The primary neuronal cell culture is a standard system for the investigation of neuronal structure and function at a high resolution. The current protocol generates relatively pure neuronal cultures with maximum reproducibility and minimal contribution of glial cells. In this method we have cultured the neurons for 3 weeks without any additional feeder cells. For cell dissociation, we have used papain only for hippocampal cells rather than trypsin, while cortical cells were dissociated by trituration without any prior enzymatic digestion. It has been observed that trypsin digestion of tissue leads to RNA degradation [20]. The cortical cells were allowed to recover from mechanical dissociation through a short incubation at 37°C in DMEM. At the same time, this step reduces the number of glial cells in the final preparation, since glial cells adhere to the plate, and a relatively pure neuronal fraction can be carefully collected in the supernatant. For biochemical methods, cells were plated directly into the wells of culture dish coated with poly-l-Lysine. For histochemical staining and live imaging, the cells were grown on glass coverslips.

Especially for biochemical assays, such as transcriptomic and proteomic analyses, it is important to control and minimize the number of other cell types in the culture, particularly the number of glial cells. In cortical cultures, we found virtually no glial cells at all during the first 2 weeks in culture and less than 5% at 21 DIV, and also in hippocampal cultures, the number of glial cells was low until DIV 14, but started to increase thereafter. This is comparable with reports using related protocols that have reported less than 5% [19] or virtually no glial cells at comparable time points [1]. It should be noted, however, that the total absence of glial cells may not be an advantage, since glia produce factors that promote the maturation and plasticity of neurons [7].

The cell lines have been the largest source for medical research due to their immortal nature. These immortal cell lines have produced variable results arising, among others, from different passage times leading to genetic drift and selection for the faster growing cells [21]. The primary cells lack the immortality factors and therefore are the best *in vitro* models for biomedical research of the post-mitotic neuronal cells. The primary cells are genetically more stable than neuronal cell lines and they maintain in culture many crucial markers and functions as seen *in vivo*. Thus, they complement *in vivo* experiments allowing for more controlled manipulation of cellular functions and processes. Once neurons are cultured, advanced molecular and biochemical studies can be easily performed. For example, successful CRISPR-cas9 gene editing has been achieved using primary neuronal cultures [22]. Furthermore, the cellular dynamics can be easily monitored through live imaging and electrophysiology. These features have established primary neurons as an essential tool for drug testing, with an additional advantage of reduced animal usage. However, variability in preparation methods reduces reproducibility of data. Therefore, we hope that the culture method for primary neurons published here could be adopted by several research groups for improved reliability and reproducibility.

## Troubleshooting tips

- The preparation of cells should be performed within no more than 2–3 h after dissecting the pups.
- Tissues should be preserved on ice all the time to prevent degradation.
- Petri dishes used are a common source of problems in the primary cell culture. We have reported here the manufacturers and plate types that have worked in our hands. Our previous experience with other manufacturers has resulted in low yield and bad quality of neurons with excessive glial contamination.
- Trituration of cortical and hippocampal tissues should be gentle without any force. Higher force results in a large number of dead cells detected in the hemocytometer counting. If the neuron quality is poor or yield is half of the average, we obtain typically using this method, these neurons should not be used for any downstream processing.

## Supplementary Material

Supplementary video S1

Supplementary video S2

Supplementary video S3

Supplementary video S4

Supplementary video S5

## Abbreviations

DIV: days *in vitro*
DMEM: Dulbecco’s modified Eagle’s medium
E17: embryonic day 17
GFAP: glial fibrillary acidic protein
Map2: microtubule-associated protein 2
NeuN: neuronal nuclei
PBS: phosphate-buffered saline
PFA: paraformaldehyde
RT: room temperature

## References

[1] Brewer G.J., Torricelli J.R., Evege E.K. and Price P.J. (1993) Optimized survival of hippocampal-neurons in B27-supplemented neurobasal (Tm), a new serum-free medium combination. J. Neurosci. Res. 35, 567–576 10.1002/jnr.490350513 8377226

[2] Banker G.A. and Cowan W.M. (1977) Rat hippocampal neurons in dispersed cell-culture. Brain Res. 126, 397–425 10.1016/0006-8993(77)90594-7 861729

[3] Bottenstein J.E. and Sato G.H. (1979) Growth of a rat neuroblastoma cell line in serum-free supplemented medium. Proc. Natl. Acad. Sci. U.S.A. 76, 514–517 10.1073/pnas.76.1.514 284369 PMC382972

[4] Brewer G.J. and Cotman C.W. (1989) Survival and growth of hippocampal-neurons in defined medium at low-density - advantages of a sandwich culture technique or low oxygen. Brain Res. 494, 65–74 10.1016/0006-8993(89)90144-3 2765923

[5] Brewer G.J. and Torricelli J.R. (2007) Isolation and culture of adult neurons and neurospheres. Nat. Protoc. 2, 1490–1498 10.1038/nprot.2007.207 17545985

[6] Seibenhener M.L. and Wooten M.W. (2012) Isolation and culture of hippocampal neurons from prenatal mice. J. Vis. Exp. 65, e3634 10.3791/3634 PMC3476399 22871921

[7] Pfrieger F.W. and Barres B.A. (1997) Synaptic efficacy enhanced by glial cells in vitro. Science 277, 1684–1687 10.1126/science.277.5332.1684 9287225

[8] Eroglu C. and Barres B.A. (2010) Regulation of synaptic connectivity by glia. Nature 468, 223–231 10.1038/nature09612 21068831 PMC4431554

[9] Banker G.A. (1980) Trophic interactions between astroglial cells and hippocampal-neurons in culture. Science 209, 809–810 10.1126/science.7403847 7403847

[10] Kaech S. and Banker G. (2006) Culturing hippocampal neurons. Nat. Protoc. 1, 2406–2415 10.1038/nprot.2006.356 17406484

[11] White L.A. and Whittemore S.R. (1992) Immortalization of raphe neurons - an approach to neuronal function-*in vitro* and *in vivo*. J. Chem. Neuroanat. 5, 327–330 10.1016/0891-0618(92)90020-Q 1524719

[12] Greene L.A. and Tischler A.S. (1976) Establishment of a noradrenergic clonal line of rat adrenal pheochromocytoma cells which respond to nerve growth-factor. Proc. Natl. Acad. Sci. U.S.A. 73, 2424–2428 10.1073/pnas.73.7.2424 1065897 PMC430592

[13] Bibel M., Richter J., Lacroix E. and Barde Y.A. (2007) Generation of a defined and uniform population of CNS progenitors and neurons from mouse embryonic stem cells. Nat. Protoc. 2, 1034–1043 10.1038/nprot.2007.147 17546008

[14] Moe M.C., Varghese M., Danilov A.I., Westerlund U., Ramm-Pettersen J., Brundin L. et al. (2005) Multipotent progenitor cells from the adult human brain: neurophysiological differentiation to mature neurons. Brain 128, 2189–2199 10.1093/brain/awh574 15958504

[15] Antila H., Autio H., Turunen L., Harju K., Tammela P., Wennerberg K. et al. (2014) Utilization of in situ ELISA method for examining Trk receptor phosphorylation in cultured cells. J. Neurosci. Methods 222, 142–146 10.1016/j.jneumeth.2013.11.001 24239780

[16] Rantamaki T., Vesa L., Antila H., Di Lieto A., Tammela P., Schmitt A. et al. (2011) Antidepressant drugs transactivate TrkB neurotrophin receptors in the adult rodent brain independently of BDNF and monoamine transporter blockade. PLoS ONE 6, e20567 10.1371/journal.pone.0020567 21666748 PMC3110188

[17] Sahu M.P., Pazos-Boubeta Y., Pajanoja C., Rozov S., Panula P. and Castren E. (2019) Neurotrophin receptor Ntrk2b function in the maintenance of dopamine and serotonin neurons in zebrafish. Sci. Rep. 9, 2036 30765816 10.1038/s41598-019-39347-3 PMC6375947

[18] Ferreira T.A., Blackman A.V., Oyrer J., Jayabal S., Chung A.J., Watt A.J. et al. (2014) Neuronal morphometry directly from bitmap images. Nat. Methods 11, 982–984 10.1038/nmeth.3125 25264773 PMC5271921

[19] , Lindholm D., Hengerer B. and Castren E. (1993) In vitro and in vivo methods for evaluating actions of cytokines on nerve growth factor production in central nervous system. Methods Neurosci. 17, 37–60

[20] Vrtacnik P., Kos S., Bustin S.A., Marc J. and Ostanek B. (2014) Influence of trypsinization and alternative procedures for cell preparation before RNA extraction on RNA integrity. Anal. Biochem. 463, 38–44 10.1016/j.ab.2014.06.017 24983903

[21] Ben-David U., Siranosian B., Ha G., Tang H., Oren Y., Hinohara K. et al. (2018) Genetic and transcriptional evolution alters cancer cell line drug response. Nature 560, 325–330 10.1038/s41586-018-0409-3 30089904 PMC6522222

[22] Swiech L., Heidenreich M., Banerjee A., Habib N., Li Y.Q., Trombetta J. et al. (2015) In vivo interrogation of gene function in the mammalian brain using CRISPR-Cas9. Nat. Biotechnol. 33, 102–106 25326897 10.1038/nbt.3055 PMC4492112

